# Intra-articular injection of collagenase in the knee of rats as an alternative model to study nociception associated with osteoarthritis

**DOI:** 10.1186/ar4436

**Published:** 2014-01-15

**Authors:** Sara Adães, Marcelo Mendonça, Telmo N Santos, José M Castro-Lopes, Joana Ferreira-Gomes, Fani L Neto

**Affiliations:** 1 Departamento de Biologia Experimental, Faculdade de Medicina da Universidade do Porto (FMUP), Alameda Prof Hernani Monteiro, Porto 4200-319, Portugal; 2 Morphysiology of the Somatosensory System Group, Instituto de Biologia Molecular e Celular (IBMC), Universidade do Porto, Porto, Portugal

## Abstract

**Introduction:**

Animal models currently used in osteoarthritis-associated pain research inadequately reproduce the initiating events and structural pathology of human osteoarthritis. Conversely, intra-articular injection of collagenase is a structurally relevant model, as it induces articular degeneration both by digesting collagen from cartilage and by causing articular instability, thereby reproducing some of the main events associated with osteoarthritis onset and development. Here, we evaluated if the intra-articular injection of collagenase can be an alternative model to study nociception associated with osteoarthritis.

**Methods:**

Osteoarthritis was induced by two intra-articular injections of either 250 U or 500 U of collagenase into the left knee joint of adult male Wistar rats. A six weeks time-course assessment of movement- and loading-induced nociception was performed by the Knee-Bend and CatWalk tests. The effect of morphine, lidocaine and diclofenac on nociceptive behaviour was evaluated in animals injected with 500 U of collagenase. Joint histopathology was scored for both doses throughout time. The expression of transient receptor potential vanilloid 1 (TRPV1) in ipsilateral dorsal root ganglia (DRG) was evaluated.

**Results:**

An increase in nociceptive behaviour associated with movement and loading of affected joints was observed after intra-articular collagenase injection. With the 500 U dose of collagenase, there was a significant correlation between the behavioural and the histopathological osteoarthritis-like structural changes developed after six weeks. One week after injection of 500 U collagenase, swelling of the injected knee and inflammation of the synovial membrane were also observed, indicating the occurrence of an early inflammatory reaction. Behavioural changes induced by the 500 U dose of collagenase were overall effectively reversed by morphine and lidocaine. Diclofenac was effective one week after injection. TRPV1 expression increased six weeks after 500 U collagenase injection.

**Conclusion:**

We conclude that the intra-articular injection of 500 U collagenase in the knee of rats can be an alternative model for the study of nociception associated with osteoarthritis, since it induces significant nociceptive alterations associated with relevant osteoarthritis-like joint structural changes.

## Introduction

Pain associated with osteoarthritis (OA) affects about 10% of the world’s population over 60 years [1] having, therefore, a high individual and socio-economic impact. It is exacerbated both by movement and loading on an affected joint [2], being the predominant reason for patients to seek medical help. Despite the relevance of pain in this disease and the wide variety of OA animal models available [3-5], only recently has research been focusing on the study of nociception in animal models of OA [6-13]. Despite inducing nociceptive responses, current models used to mimic OA pain present significant disadvantages, mainly in what concerns the mechanisms of OA onset and development [14]. Ideally, it would be desirable that an experimental model could reproduce as closely as possible the different features of a disease. It seems reasonable to state that the development of chronic pain in OA should not be dissociated from the structural articular changes that occur during the onset and progression of OA. A model that comprises both the reproduction of the initiating events and joint tissue pathology observed in human OA as well as the induction of relevant nociceptive responses that mimic patients’ main complaints, such as increased nociception due to movement and loading on the affected joint, would certainly be a clinically-relevant model for the study of OA pain.

The intra-articular (i.a.) injection of collagenase is an established model of OA that has been predominantly used to study the mechanisms underlying structural joint damage [15-17]. Histopathological alterations of the knee joint very similar to those observed in human OA have been described for this model, particularly in mice [18,19] and rabbits [20], as well as in the lumbar facet joint of rats [21].

Collagenase induces the degeneration of the articular cartilage by directly digesting collagen from the extracellular matrix of cartilage [20], and as a consequence of articular instability due to increased joint laxity [22]. Changes in other articular structures from which pain may originate, such as the subchondral bone and synovial membrane [17,19,20], are also observed, thereby reproducing some of the main features associated with OA onset and development in humans [23-28].

The relevance of collagen degradation and of articular instability as features of OA, as observed in the clinical setting, leading to histopathological alterations that highly correlate with those described for human OA [29], make the i.a. injection of collagenase a clinically-relevant model for the study of OA structural pathophysiology, and a promising alternative to study OA-associated pain.

Therefore, we aimed at determining whether i.a. collagenase injection in the knee could also be a good model to study OA-associated nociception in rats by evaluating nociception induced by movement and loading on the affected joint. We also tested the efficacy of morphine in reversing behavioural changes with the goal of validating them as being nociception-related, as well as the efficacy of a local anaesthetic intra-articularly injected in the knee, in order to demonstrate that the nociceptive behaviours observed are indeed originated in the knee joint. Furthermore, since there seemed to be an inflammatory reaction occurring after the injection of collagenase, we also tested the effect of the non-steroidal anti-inflammatory drug (NSAID) diclofenac in reversing the nociceptive changes observed. Additionally, to determine if changes in the sensory innervation of the knee could also be observed in this model, we evaluated, in the dorsal root ganglia (DRG) from control and collagenase-injected animals, the expression of the transient receptor potential vanilloid 1 (TRPV1), an ion channel whose role in nociception is widely reported [30-33].

## Methods

### Animals

Adult male Wistar rats (230 ± 30 g, Charles River Laboratories, Barcelona, Spain) were housed in groups of three, with water and food *ad libitum*, at a constant temperature of 22°C and controlled lighting (12 h light/12 h dark cycle). A total of 99 animals were used in this study: 72 for behavioural testing and 27 for the pharmacological evaluation, 5 per drug (15 in total) and 4 per control (12 in total).

Experimental procedures were performed in accordance with the ethical guidelines for the study of experimental pain in conscious animals [34], and the European Communities Council Directive 86/609/EEC amended by the Directive 2003/65/CE and were approved by the Ethical Committee for health of Hospital de São João, Porto, Portugal.

### Osteoarthritis induction

Under brief isoflurane anaesthesia (5% isoflurane for anaesthesia induction, 2% for maintenance), an intra-articular injection was performed with the use of a Hamilton syringe, with a 26 G needle inserted through the patellar ligament into the joint space of the left knee. Animals received two injections, one on day 0 and another on day 3, with 25 μl of either sterile saline (control group), 250 U or 500 U of type II collagenase from *Clostridium histolyticum* (Sigma-Aldrich, St.Louis, MO, USA) dissolved in saline and filtered through a 0.22 μm membrane [20]. Animals were randomly assigned to each group before the first injection. Doses of collagenase were chosen based on a previous study that assessed nociceptive alterations in rats [35] injected with 500 U of collagenase. We tested the same dose and a smaller one, to evaluate if similar changes could be obtained with a smaller amount of exogenous collagenase.

### Behavioural testing

Animals injected with saline, 250 U or 500 U collagenase (72 animals; n = 24/treatment) were randomly divided in groups that were sacrificed at different time-points: one, two, four and six weeks after the first injection (n = 6/group/treatment). Movement-induced nociception was evaluated by the Knee-Bend and CatWalk tests [13] on Day 0 (before the first injection), and at one, two, three, four, five and six weeks after the first injection, until each group’s endpoint. Testing was performed blindly, always by the same experimenter. The Knee-Bend test consists in counting the squeaks and/or struggle reactions in response to five alternate flexions and extensions of the knee joint, performed within the physiological limits of knee flexion/extension. The score of the test was determined by the type of reaction to each movement of the joint as follows: 0 - no responses to any kind of movement of the joint; 0.5 - struggle to maximal flexion/extension; 1 - struggle to moderate flexion/extension or vocalizations to maximal flexion/extension; 2 - vocalizations to moderate flexion/extensions. A maximal extension corresponds to placing the knee joint in an 180° angle; a moderate extension corresponds to an angle between 120° and 150°, approximately; a moderate flexion corresponds to an angle between 45° and 75°, approximately; a maximal flexion corresponds to totally bending the knee joint (corresponding approximately to an angle of 30°). The sum of the animal’s reactions, giving maximal values of 20, represents the Knee-Bend score, an indication of the animal’s movement-induced nociception. The contralateral knee was always tested first, in order to avoid an increase in the contralateral score arising from the manipulation of the injected knee. Results for both ipsilateral and contralateral knees were presented.

For the CatWalk test, animals were placed in a glass platform illuminated such as to reflect light only at the points of contact of the paw with the surface, resulting in a bright image of the paw print. Videos were acquired by a camera placed under the platform. The signal intensity depended on the paw area in contact with the platform and increased with the pressure applied by the paw. Random frames of the videos were analysed: three pairs of frames (one for each hind paw) with the animal walking and three frames with the animal standing still. For each hind paw, the number and intensity of pixels above a defined threshold were quantified (Image J 1.37, [36]) to determine the total paw print intensity (mean pixel intensity × number of pixels), allowing the comparison of the area/pressure applied by each paw. Results were expressed as the percentage of the total ipsilateral paw print intensity (%TIPPI) in the total intensity of both paw prints.

The CatWalk test was always performed prior to the Knee-Bend test, to minimize the effect of manipulation of the affected knee joint on the animals’ gait.

### Joint swelling

Knee diameters were measured to infer joint swelling as an indicator of inflammation resulting from i.a. injection of collagenase or saline. The diameter of both knees was measured with a manual calliper. Results were presented as the difference in knee diameter (ipsilateral-contralateral).

### Tissue processing

At one, two, four and six weeks after the first injection of saline, 250 U or 500 U of collagenase, animals were perfused with 4% paraformaldehyde (n = 6/group/treatment). Their ipsilateral DRG from lumbar segments L3, L4 and L5 were dissected, post-fixed in the same fixative (four hours) and kept in 30% sucrose with 0.01% sodium azide. DRG were serially sliced in 12 μm sections using a cryostat, with every 10^th^ section collected in the same slide. DRG were always cut longitudinally yielding 8 to 10 sections from each, on average. The injected knees were also dissected, post-fixed for 72 hours and then decalcified for 8 hours in a buffer containing 7% AlCl_3_, 5% HCOOH and 8.5% HCl. The joints were then washed in 0.1 M phosphate buffer pH 7.2, and kept in 30% sucrose with 0.01% sodium azide until they were cut. Joints were cut into two approximately equal halves along the medial collateral ligament in the frontal plane. Three 10 μm frozen sections were cut from each half at 200 μm steps using a cryostat.

### Histopathology

Knee joint sections were stained either with Haematoxylin and Eosin or by the Fast Green and Safranin-O method to evaluate the extent of the histopathological lesions. Slides were mounted with Eukitt (Kindler GmbH & Co., Baden-Württemberg, Germany) and images acquired with an Axioskop-40 microscope equipped with an AxioCam-MRc5 camera (Carl Zeiss MicroImaging GmbH, Jena, Germany).

Histological scoring was performed for the medial tibial plateau (MTP) of the three most severely affected sections based on the Osteoarthritis Research Society International (OARSI) recommendations for histological assessment of osteoarthritis in the rat [37]. Using an image analysis software (Image Pro-plus 5.0, Media Cybernetics, Rockville, MD, USA), the following parameters were evaluated, as fully described by Gerwin and colleagues [37] and briefly described here:

● Cartilage matrix loss width (CMLW). The width of the areas of complete cartilage matrix loss was measured along the surface (0% depth), the midzone (50% depth) and tidemark (100% depth).

● Total cartilage degeneration width (TCDW). The total width of the area of articular cartilage affected by any type of degenerative change was measured.

● Significant cartilage degeneration width (SCDW). The width of the cartilage in which 50% or greater of its thickness (from surface to tidemark) was compromised was measured.

● Zonal depth ratio (ZDR) of lesions. The MTP was divided into three zones in order to evaluate the pathology of different load-bearing areas, with zone 1 being the medial edge of the joint, zone 2 the central area of the MTP, and zone 3 adjacent to the cruciate ligaments. The depth of cartilage degeneration was taken at the midpoint in each of the three zones across the tibial surface. The lesion depth ratio was calculated by dividing the depth of the area of degeneration by the thickness of the cartilage, from projected cartilage surface to tidemark.

● Cartilage degeneration score (CDS). The MTP was divided into the three different load-bearing zones, as described for ZDR. This parameter was an evaluation of overall cartilage pathology with chondrocyte loss being the primary determinant of the score. Cartilage degeneration in each zone was scored using the following criteria: 0 - no degeneration; 1 - minimal degeneration, 5 to 10% of the total projected cartilage area affected by matrix or chondrocyte loss; 2 - mild degeneration, 11 to 25% affected; 3 - moderate degeneration, 26 to 50% affected; 4 - marked degeneration, 51 to 75% affected; 5 - severe degeneration, greater than 75% affected.

● Osteophytes score (OS). The largest osteophyte in each section was measured at its thickest point. Based on that measurement a grade was assigned: 0 - proliferative changes, <200 μm; 1 - small, 200 to 299 μm; 2 - moderate, 300 to 399 μm; 4 - large, 400 to 499 μm; 5 - very large, >500 μm.

● Synovial membrane inflammation score (SMIS). The number of synovial lining cell layers, the proliferation of subsynovial tissue, and the infiltration of inflammatory cells were evaluated according to the following scale: 0 - no changes (one to two layers of synovial lining cells); 1 - increased number of lining cell layers (three to four layers) or slight proliferation of subsynovial tissue; 2 - increased number of lining cell layers (three to four layers) and/or proliferation of subsynovial tissue; 3 - increased number of lining cell layers (more than four layers) and/or proliferation of subsynovial tissue and infiltration of few inflammatory cells; 4 - increased number of lining cell layers (more than four layers) and/or proliferation of subsynovial tissue, infiltration of large number of inflammatory cells.

● Medial joint capsule repair (MJCR). The thickness of the medial joint capsule was measured.

● Growth plate thickness (GPT). Growth plate thickness was measured medially and laterally (two measures/joint), midway between the centre of the physis and the medial (medial measurement) or lateral (lateral measurement) margin. Since no differences were observed between the medial and lateral measurements, results were presented as an average of both measurements.

### Immunofluorescence for TRPV1

Slides containing every 10^th^ section of ipsilateral L3, L4 or L5 DRG of animals sacrificed one or six weeks after collagenase or saline injection were used for immunofluorescence reactions against TRPV1. DRG sections were rinsed in 0.1 M phosphate-buffered saline (PBS; pH 7.4), followed by PBS + 0.3% triton X (PBST), and incubated in PBST with 10% normal serum (1 h 30). Sections were then incubated with guinea-pig anti-TRPV1 diluted in PBST + 2% normal serum (1:250, Chemicon, Temecula, CA, USA), overnight at room temperature. After thorough PBST washing, sections were incubated with Alexa Fluor 568 donkey anti-guinea-pig secondary antibody diluted in PBST + 2% normal serum (1:2,000, one hour, room temperature, Molecular Probes, Eugene, OR, USA). Slides were then rinsed in PBST followed by PBS, mounted with Prolong Gold Antifade mounting medium (Molecular Probes) and cover-slipped. Negative controls were performed by following the same procedure without the primary antibody. DRG sections were viewed using a Zeiss Imager.Z1 fluorescence microscope (Carl Zeiss MicroImaging GmbH, Jena, Germany) and images were acquired using an AxioCam MRm with AxioVision 4.6 software (Carl Zeiss MicroImaging GmbH, Jena, Germany) at a 100x magnification. All neurons and all labelled cells were counted in one slide containing every 10^th^ section of each DRG. Data were presented as the percentage of neurons expressing TRPV1 in L3, L4 and L5 DRG.

### Pharmacological experiments

A pharmacological evaluation was performed in different groups of animals at one, two, four and six weeks after 500 U collagenase injection using the opiate morphine, the local anaesthetic lidocaine and the NSAID diclofenac. Baseline values of nociception were determined before drug administration (t = 0 minutes). To confirm that the behavioural responses of OA animals in the CatWalk and Knee-Bend tests were induced by nociception, we evaluated if they could be reversed by a single administration of a non-sedative dose of morphine [7]. Morphine (6 mg/mL in saline, Labesfal, Lisboa, Portugal) was subcutaneously (s.c.) injected in the upper dorsum in a dose of 6 mg/Kg [7,38] (n = 5), and drug effects on both tests were assessed 30, 60, 90, 120 and 180 minutes after administration. Control animals were also injected with 500 U of collagenase and received a s.c. injection of saline (n = 4). Behavioural assessment was performed after 30, 60 and 120 minutes. To determine if nociception in this model originates from knee articular tissues, we intra-articularly injected the local anaesthetic lidocaine in the knee of rats injected with 500 U of collagenase. Lidocaine (100 mg/mL in saline, Sigma) was administered in a dose of 5 mg, in a volume of 50 μL [7,38] (n = 5). Drug effects on both tests were assessed after 10, 20 and 30 minutes. Control animals received an intra-articular injection of saline (n = 4). To assess if inflammation occurs during the onset and/or development of the model, we evaluated the effect of the NSAID diclofenac. Diclofenac (15 mg/mL in bidistilled water, Sigma) was administered *per os* (p.o.) in a dose of 30 mg/Kg [7,38] (n = 5), and drug effects on both tests were assessed 30, 60, 90 and 120 minutes after drug administration. Control animals were also injected with 500 U of collagenase and received a p.o. administration of bidistilled water (n = 4). Data are presented as the percentage of changes in relation to baseline values for the time-point of peak effect for each drug and nociceptive test. Animals were randomly assigned to each group and testing was performed blindly. Post-administration time-points for behavioural assessment were determined in preliminary experiments.

### Statistics

Animals were randomly assigned to each group. Results are presented as mean ± SEM. The normality of all data was assessed by the Kolmogorov-Smirnov test. Behavioural data for movement induced-nociception in control, 250 U and 500 U collagenase-injected groups were analysed by two-way ANOVA, for factors time and group, followed by Bonferroni *post-hoc* test for multiple comparisons between groups; ipsilateral vs. contralateral comparisons in the Knee-Bend test were performed likewise. The pharmacological data were analysed by repeated measures ANOVA followed by Dunnett’s *post-hoc* test. For the histological assessment, measured parameters were analysed by one-way ANOVA followed by Tukey *post-hoc* test; scored parameters were analysed by the Kruskal-Wallis test with Dunn’s *post-hoc* test. Pearson’s correlation analysis was performed for data from control and 500 U collagenase-injected animals. The expression of TRPV1 was evaluated by the Mann–Whitney test. A *P*-value <0.05 was accepted as statistically significant.

## Results

### Joint swelling

**Figure 1:**
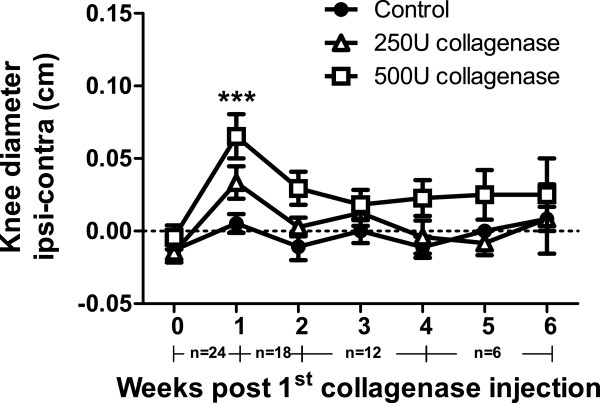
**Inflammation index.** Joint swelling assessed by measuring knee diameters (difference between ipsilateral and contralateral knees). Collagenase-injected animals present increased knee diameters one week after injection. The number of animals per group at each time-point is indicated below the graphs. An initial number of 24 animals was used in each group. Animals were then sacrificed at different time-points for histopathology (one, two, four and six weeks; n = 6 per dose and per time-point). Mean ± SEM, two-way ANOVA followed by Bonferroni *post-hoc* test for comparisons between groups at each time-point. ***P <0.001, for comparisons between control and the 500 U collagenase group.

Signs of inflammation were detected after collagenase injection, denoted by a dose dependent increase in the difference between the ipsilateral and contralateral knee diameter at one week, significant only for the 500 U dose (*P* <0.001, Figure 1), with ipsilateral joint diameters returning to contralateral values thereafter.

### Nociceptive behaviour

**Figure 2:**
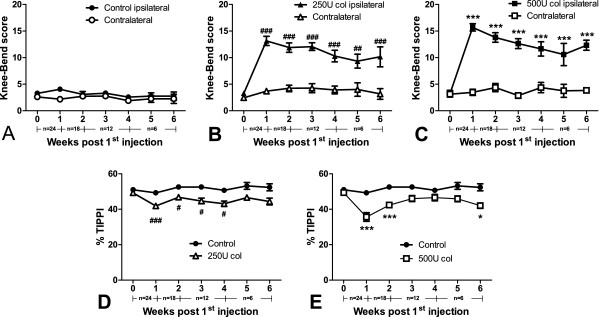
**Nociceptive behaviour.** Nociception induced by movement and loading in control and collagenase-injected rats evaluated by the Knee-Bend and CatWalk tests. Knee-Bend score **(A-C)** is presented for both ipsilateral and contralateral knees of saline-injected control rats **(A)**, and rats injected with 250 U **(B)** or 500 U **(C)** of collagenase. CatWalk data **(D, E)** are expressed as the percentage of total ipsilateral paw print intensity (%TIPPI). Collagenase-injected animals present increased movement- and loading-induced nociception. The number of animals per group at each time-point is indicated below the graphs. An initial number of 24 animals was used in each group. Animals were then sacrificed at different time-points for histopathology (one, two, four and six weeks; n = 6 per dose and per time-point). Mean ± SEM, two-way ANOVA followed by Bonferroni *post-hoc* test for comparisons between groups at each time-point. #*P* <0.05, ###*P* <0.001, for comparisons between control and the 250 U collagenase group; *P <0.05, ***P <0.001, for comparisons between control and the 500 U collagenase group.

The Knee-Bend (Figure 2A-C) and CatWalk (Figure 2D-E) tests were used to evaluate loading- and movement-induced nociception following the i.a. injection of different doses of collagenase. In both tests and at all time-points, saline-injected control animals showed behavioural responses similar to those observed prior to the injection (day 0; 3.3 ± 0.3 for the ipsilateral Knee-Bend score; 51.0 ± 0.7% for the CatWalk). In collagenase-injected animals, behavioural changes were time-dependent, with the highest increase in nociception being observed one week after the first collagenase injection, and remaining higher than on day 0 or in control animals throughout the whole period of analysis for both collagenase doses (Figure 2). For the 250 U, ipsilateral Knee-Bend scores (Figure 2B) increased from 3.3 ± 0.4 on day 0 to 13.1 ± 0.9 on week 1 (*P* <0.001), slightly decreasing throughout time, but remaining significantly different from the contralateral knee and control animals until the sixth week (10.2 ± 1.7, *P* <0.001). For the 500 U dose, ipsilateral Knee-Bend scores (Figure 2C) increased from 3.2 ± 0.6 on day 0 to 15.7 ± 0.7 on week 1 (*P* <0.001), slightly decreasing throughout time, but remaining significantly different from the contralateral knee and control animals until the sixth week (12.3 ± 1.0, *P* <0.001). The increase in the ipsilateral Knee-Bend score for the 500 U dose at week 1 was significantly higher than the increase observed with the 250 U dose (*P* <0.05). The values obtained with the 500 U remained higher than those with the 250 U throughout time, although differences were not statistically significant. Likewise, in the CatWalk test, the %TIPPI showed a similar pattern for both doses, being always below the values observed on day 0 and in control animals, although not always reaching statistical significance (Figure 2D). Thus, for the 250 U dose the %TIPPI decreased from 49.3 ± 1.0 on day 0 to 41.9 ± 1.4 at week 1 (*P* <0.001), when the lowest values were observed, slightly increasing until the sixth week to a value of 44.4 ± 1.9, not significantly different from control animals. For the 500 U dose (Figure 2E) the %TIPPI decreased from 49.4 ± 0.8 on day 0 to 35.6 ± 2.4 at week 1 (*P* <0.001), when the lowest values were observed, and increased from then until week 4 (46.6 ± 1.9, n.s.), decreasing again until the sixth week (42.0 ± 1.5, *P* <0.05). As for the Knee-Bend test, the decrease in the %TIPPI for the 500 U dose at week 1 was significantly higher than for the 250 U dose (*P* <0.01), with 500 U values remaining lower at late time-points but not significantly different.

### Pharmacological evaluation

**Figure 3:**
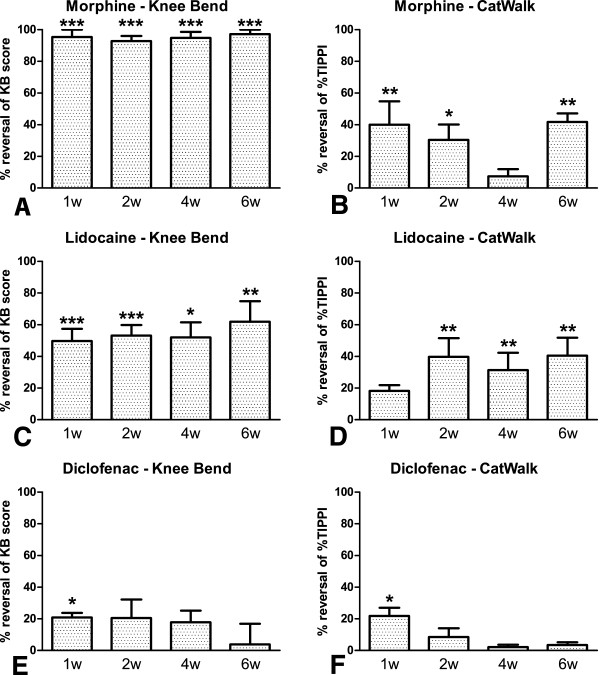
**Pharmacological evaluation.** Effect of morphine (6 mg/Kg, s.c., **A, B**), lidocaine (5 mg, i.a., **C, D**) or diclofenac (30 mg/Kg, p.o., **E, F**) on movement- and loading-induced nociception assessed by the Knee-Bend **(A,C,E)** and the CatWalk **(B,D,F)** tests, at one, two, four and six weeks after injection of 500 U of collagenase. **A**: Morphine effectively reversed the Knee-Bend scores at all time-points of disease progression. **B**: Morphine was effective at one, two and six weeks of disease progression. **C**: Lidocaine effectively reversed the Knee-Bend scores at all time-points of disease progression. **D**: Lidocaine was effective at two, four and six weeks of disease progression. **E**: Diclofenac effectively reversed the Knee-Bend scores one week after injection. **F**: Diclofenac was effective one week after injection. Mean ± SEM. Repeated measures ANOVA followed by Dunnett’s *post-hoc* test; *P <0.05, **P <0.01, ***P <0.001.

The effect of an acute administration of morphine, lidocaine and diclofenac on loading- and movement-induced nociception was evaluated at one, two, four and six weeks after injection of 500 U of collagenase (Figure 3), since this was the dose that induced nociceptive changes that better correlated with relevant histopathological alterations. All animals showed increased nociceptive behaviour before drug or vehicle administration (t = 0 minutes, baseline). The effect of a s.c. injection of saline, a p.o. administration of bidistilled water or an intra-articular administration of saline was evaluated in animals injected with 500 U collagenase to monitor the vehicle’s effects. We obtained similar results to those already published by our group for the monoiodoacetate (MIA) model of OA [38], with no changes being observed (data not shown).

Morphine was highly effective at all time-points of disease progression in the Knee-Bend test, fully reversing the nociceptive behaviour (Figure 3A, B). The maximal effect was observed between 30 and 90 minutes after injection, starting to revert thereafter. Thirty minutes after administration, morphine significantly reduced the ipsilateral Knee-Bend score (Figure 3A) by 95 ± 5% at week 1 (*P* <0.001), 93 ± 3% at week 2 (*P* <0.001), 95 ± 4% at week 4 (*P* <0.001) and 97 ± 3% at week 6 (*P* <0.001). In the CatWalk test (Figure 3B), morphine was effective one, two and six weeks after collagenase injection and maximal effect was observed from 60 to 90 minutes. Sixty minutes after administration, the %TIPPI increased by 40 ± 15% at week 1 (*P* <0.01), 30 ± 10% at week 2 (*P* <0.05), 7 ± 5% at week 4 (n.s.) and 42 ± 5% at week 6 (*P* <0.01).

Lidocaine was also effective in diminishing the nociceptive behaviour (Figure 3C, D) at all time-points. The maximal effect was observed 10 minutes after injection, starting to revert thereafter. In the Knee-Bend test (Figure 3C), lidocaine significantly reduced the ipsilateral Knee-Bend score by 50 ± 8% at week 1 (*P* <0.001), 53 ± 7% at week 2 (*P* <0.001,), 52 ± 9% at week 4 (*P* <0.05) and 62 ± 13% at week 6 (*P* <0.01). In the CatWalk test (Figure 3D), lidocaine increased the %TIPPI by 18 ± 4% at week 1, although this was not statistically significant, 40 ± 12% at week 2 (*P* <0.01), 31 ± 11% at week 4 (*P* <0.01) and 41 ± 11% at week 6 (*P* <0.01).

Diclofenac, on the other hand, was only significantly effective at week 1 (Figure 3E, F). Its maximal effect occurred 30 minutes after injection with a decrease of 21 ± 3% in the Knee-Bend score (*P* <0.05, Figure 3E) and an increase of 22 ± 5 in the %TIPPI in the CatWalk test (*P* <0.05, Figure 3F).

### Histopathology

**Figure 4:**
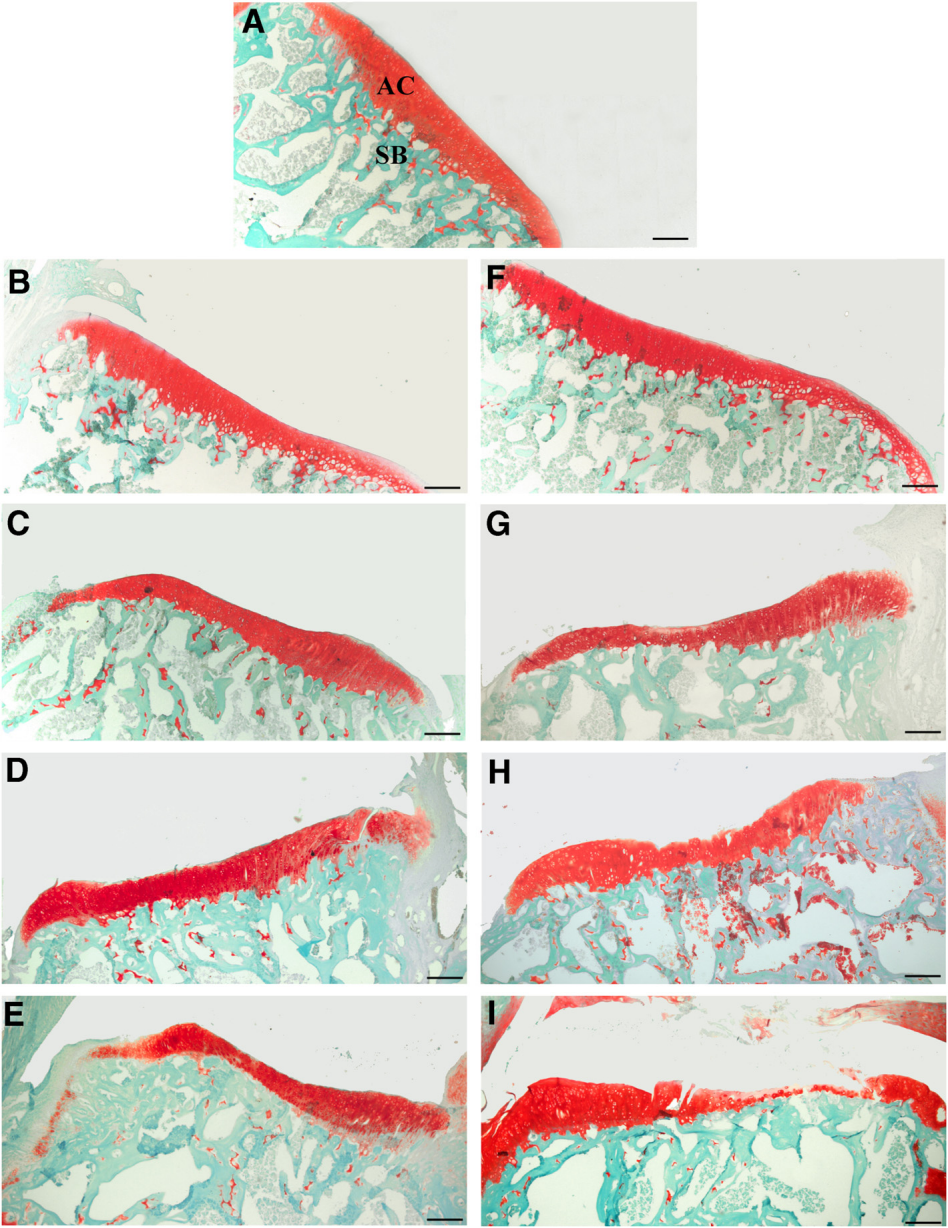
**Progression of histopathological changes in knee joint sections stained with Fast Green and Safranin O.A**: Control animals show unaltered articular cartilage (AC) and subchondral bone (SB). Animals injected with 250 U collagenase **(B, C, D, E)** show increased thinning of the articular cartilage throughout time, along with focal chondrocyte disorganization. Animals injected with 500 U **(F, G, H, I)** show signs of OA onset at week 4 **(H)** that further develop until week 6 **(I)** when extensive damage of the articular cartilage is observed. Bar: 50 μm.

**Figure 5:**
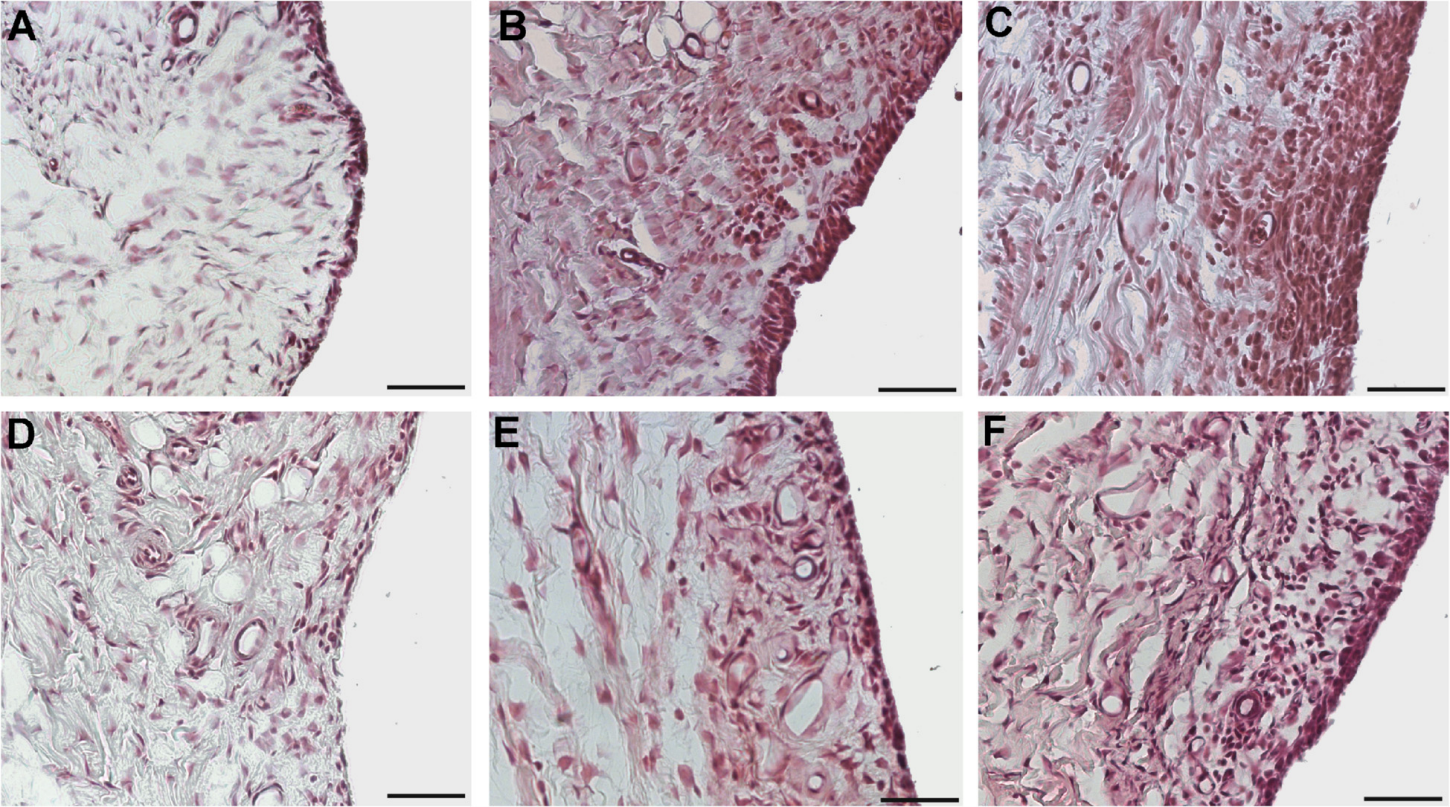
**Synovial membrane histopathology.** Images representative of the synovial membrane inflammation one week **(A-C)** and six weeks **(D-F)** after injection of saline **(A,D)**, 250 U **(B, E)** or 500 U of collagenase **(C, F)**. An increased number of synovial lining cell layers, proliferation of subsynovial tissue and infiltration of inflammatory cells are observed. Changes were most pronounced with the 500 U dose, one week after injection, receding thereafter. Bar: 50 μm.

**Figure 6:**
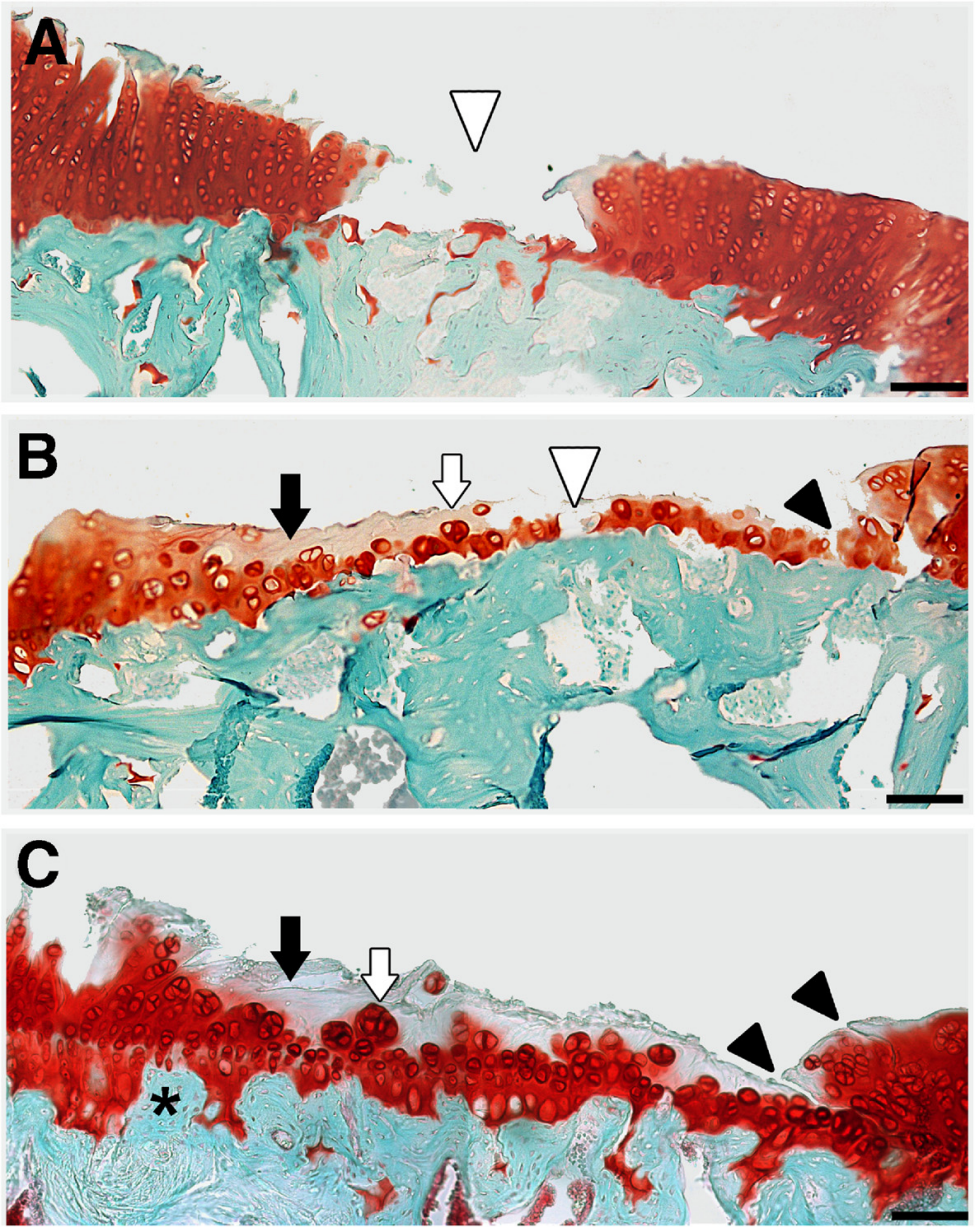
**Articular cartilage degeneration six weeks after the injection of 500 U of collagenase.** Extensive cartilage degeneration, comprising areas of marked erosion with exposure of the subchondral bone (white arrowhead; **A, B**), loss of proteoglycan staining and cell depletion (black arrow; **B**, **C**), chondrocyte clustering and hypertrophy (white arrow; **B**, **C**), fissures (black arrowhead; **B**, **C**) and tidemark undulation (*; **C**). Bar: 50 μm.

No histopathological damage in the knee joint could be found in saline-injected control animals at any time-point studied (Figure 4A). In contrast, dose- and time-dependent alterations were observed in collagenase-injected rats, which were always more pronounced in the MTP (authors’ observation, not shown). At week 1, a slight superficial loss of proteoglycan staining was observed in animals injected with both doses of collagenase (Figure 4B, F), as well as a high degree of a dose-dependent synovial inflammation (Figure 5B, C). Two weeks after collagenase injection, there was a dose-dependent thinning of the articular cartilage (Figure 4C, G), accompanied by focal chondrocyte disorganization, for the 250 U dose (Figure 4C), and by chondrocyte clustering and hypertrophy for the 500 U dose (Figure 4G). Synovial inflammation, although less pronounced than at week 1, was also observed, particularly with the 500 U dose. At week 4, some erosion of the articular surface was observable with the 250 U dose (Figure 4D). With the 500 U dose further signs of OA development were observed, such as the occurrence of small fissures and superficial erosion of the articular cartilage, along with chondrocyte clustering and hypertrophy (Figure 4H). Synovial inflammation was reduced for both doses and some small osteophytes could be observed. After six weeks, the 250 U dose induced loss of proteoglycan staining, focal chondrocyte disorganization, irregularity of the articular surface, and areas of cartilage matrix loss (Figure 4E). With the 500 U dose, clear signs of OA development were observed: extensive damage of the articular cartilage (Figures 4I and 6); areas of erosion with deep cartilage matrix loss and focal areas of exposure of the subchondral bone (Figure 6A, B); loss of proteoglycan staining, chondrocyte loss (Figure 6B, C) or chondrocyte clustering and hypertrophy and fissures of the cartilage (Figure 6B, C). Cartilage collapse into the epiphysis was also extensively observed (Figure 6C) and osteophytes were also found; some signs of synovial inflammation could also be observed (Figure 5E, F), although much less pronounced than at one and two weeks.

### Histopathological scoring

**Figure 7:**
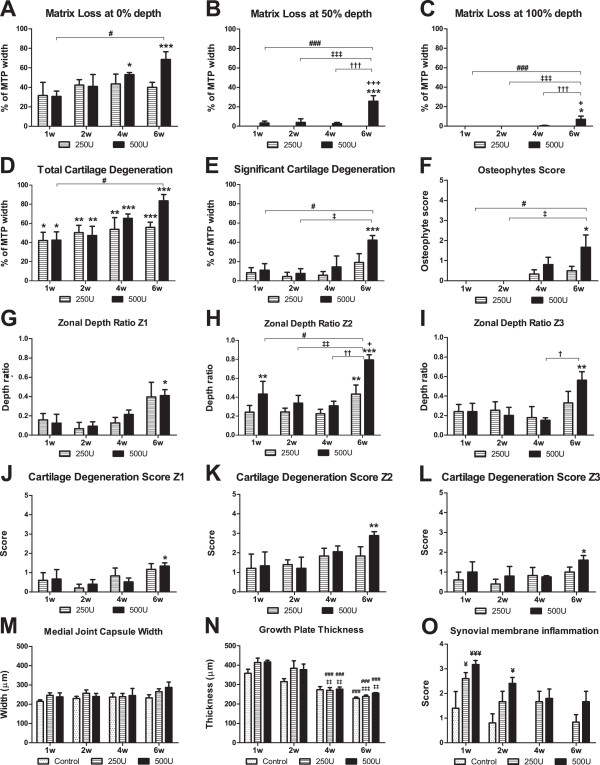
**Histopathological scoring.** Histopathological assessment of cartilage degenerative changes in the medial tibial plateau (MTP) of control animals and animals injected with 250 or 500 U collagenase, one, two, four and six weeks after injection. The following parameters were evaluated: cartilage matrix loss width (CMLW) measured along the surface (0% depth, **A**), the midzone (50% depth, **B**) and tidemark (100% depth, **C**); total cartilage degeneration width (TCDW, **D**); significant cartilage degeneration width (SCDW, **E**); osteophyte score (OS, **F**); zonal depth ratio (ZDR) of lesions at zone 1 (at the medial edge of the joint, **G**), zone 2 (at the centre of the MTP, **H**) and zone 3 (adjacent to the cruciate ligaments, **I**); cartilage degeneration score (CDS) at zones 1 (**J**), 2 (**K**) and 3 (**L**); synovial membrane inflammation score (SMIS, **M**); medial joint capsule width (MJCW, **N**); growth plate thickness (GPT, **O**). Mean ± SEM. Measured parameters were analysed by one-way ANOVA followed by Tukey *post-hoc* test; scored parameters were analysed by the Kruskal-Wallis test with Dunn’s *post-hoc* test. *P <0.05, **P <0.01, ***P <0.001, for comparisons between collagenase injected and control animals within each time point; ^**+**^*P* <0.05, ^**+++**^*P* <0.001, for comparisons between the 250 and 500 U groups within each time-point; ^#^*P* <0.05, ^###^*P* <0.001, for comparisons between week 1 and week 6 within each group; ^**‡**^*P* <0.05, ^**‡‡**^*P* <0.01, ^**‡‡‡**^*P* <0.001, for comparisons between week 2 and week 6 within each group; ^**†**^*P* <0.05, ^**††**^*P* <0.01, ^**†††**^*P* <0.001, for comparisons between week 4 and week 6 within each group; ^**¥**^*P* <0.05, ^**¥¥**^*P* <0.01, ^**¥¥¥**^*P* <0.001, between collagenase injected and control animals from a different time point.

All measurements are presented in Figure 7. In control animals, changes were barely observed, being shown only when they were measurable. In collagenase-injected rats, there was a continuous and dose-dependent progression of OA evidenced by a higher degree of joint destruction for the 500 U dose and throughout the time of OA development for all parameters scored. Overall, there was a higher loss of cartilage matrix after six weeks and with the 500 U dose, as shown by the CMLW parameter. CMLW at the surface (0% depth, Figure 7A) was significantly enhanced both at four (*P* <0.05) and six weeks (*P* <0.001), reaching an average of 69 ± 8% of the superficial cartilage showing matrix loss. Deeper areas of the cartilage were only affected significantly six weeks after the injection of 500 U collagenase, as indicated by CMLW at the midzone (50% depth, Figure 7B), with 26 ± 6% (*P* <0.001) of the MTP width losing at least 50% of its depth. Exposure of the subchondral bone was observed in some sections after six weeks with the 500 U dose (Figure 6), as reflected in the CMLW at the tidemark (100% depth, Figure 7C) that showed that an average of 7 ± 3% of the cartilage width was lost. The TCDW measurement (Figure 7D) showed that cartilage degeneration, independent of the loss of the extracellular matrix, was already observable after one week for both doses, but continuously developed until the sixth week, when 84 ± 7% (*P* <0.001) of the cartilage width of animals injected with 500 U collagenase was affected by changes that included loss of proteoglycan staining, chondrocyte clustering and hypertrophy or chondrocyte death, and fissures (Figure 6). SCDW, which measures the areas where at least 50% of the cartilage depth is affected by any type of degenerative change (Figure 7E), showed the increased depth of cartilage alterations throughout time, being significant only at week 6 for the 500 U dose, when 42 ± 5% of its width was affected (*P* <0.05). This was further reinforced by the ZDR of lesions (Figure 7G-I), which showed that the average ratio of the cartilage depth affected significantly increased at six weeks. This parameter also showed that there is a different degree of destruction in different load bearing areas. In fact, the ZDR of lesions was higher in zone 2 (Figure 7H), where significant differences were already observed at week 1 for the 500 U dose (*P* <0.01), further increasing to 0.79 ± 0.06 at week 6 (*P* <0.001). Zone 3, next to the cruciate ligaments, showed a ZDR of 0.56 ± 0.09 at week 6 (*P* <0.01, Figure 7I), whereas in zone 1, the most medial area of the MTP, the ZDR was 0.41 ± 0.06 at week 6 (*P* <0.05, Figure 7G). The CDS, which takes into account the percentage of cartilage affected by any type of change, again showed the different degree of destruction at different load bearing areas (Figure 7J-L). Changes were significant only after six weeks and only for the 500 U dose of collagenase, and most pronounced in zone 2, where the average OA score was 2.9 ± 0.25 (out of 5, *P* <0.05, Figure 7K). The development of osteophytes was observable for both doses from week 4 onwards and was more pronounced six weeks after 500 U collagenase injection, when some large osteophytes could be observed (Figure 7F). Synovial inflammation (Figures 5 and 7M) was observed in control animals at one and two weeks, probably as a reaction to the intra-articular injection of saline. Collagenase-injected animals, however, showed a much higher degree of synovial inflammation at week 1, particularly for the 500 U dose, which diminished throughout time. No differences were observed in the medial joint capsule (Figure 7N). The growth plate thickness diminished over time, similarly to what was observed in controls (Figure 7O).

### Correlation analysis

The correlation analysis between the behavioural data and the histopathological score as well as the knee diameter, for both doses, at weeks 1 and 6, is shown in Table 1. This correlation was performed with the data from the animals sacrificed at each time-point (n = 6/group). Noteworthy results include the significant correlation observed between the Knee-Bend score and the knee diameter (*P* <0.01) one week after injection for both doses. The CatWalk test did not correlate with the knee diameter. Some correlations were found for the Knee-Bend in the 250 U dose at both time points, namely with the TCDW and the ZDR for zones 2 and 3. The CatWalk showed no correlations for this dose at both time-points. For the 500 U dose, a larger number of correlations could be observed. At week 1, a correlation between both tests and the CMLW 0% was observed, most significant for the Knee-Bend test (*P* <0.001). This test also correlated with the TCDW (*P* <0.01), the ZDR at zone 2 (*P* <0.01) and with the synovial inflammation (*P* <0.05). At week 6, there were significant correlations between the Knee-Bend score and most of the histopathological parameters analysed (Table 1), namely with the CMLW at 0% depth (*P* <0.01), the TCDW (*P* <0.01), the SCDW (*P* <0.01), the ZDR at zones 2 (*P* <0.01) and 3 (*P* <0.05) and the CDS at zones 1 (*P* <0.01) and 2 (*P* <0.01) and the SMIS (*P* <0.01). CatWalk data also significantly correlated with the CMLW at 0% depth (*P* <0.05), the TCDW (*P* <0.05) and the CDS at zone 2 (*P* <0.05).

**Table 1:** Correlation analysis

**Pearson correlation coefficient**								
	**One week**				**Six weeks**			
	**250 U**		**500 U**		**250 U**		**500 U**	
**vs**	**KB**	**CW**	**KB**	**CW**	**KB**	**CW**	**KB**	**CW**
**Knee diameter**	0.768	−0.137	0.791	0.136	−0.017	0.564	−0.152	0.350
	**	-	**	-	-	-	-	-
**CMLW 0%**	0.474	−0.389	0.929	−0.732	0.764	−0.436	0.808	−0.710
	-	-	***	*	*	-	**	*
**CMLW 50%**	-	-	0.506	−0.495	-	-	0.569	−0.609
	-	-	-	-	-	-	-	-
**CMLW 100%**	-	-	-	-	-	-	0.456	−0.221
	-	-	-	-	-	-	-	-
**TCDW**	0.674	−0.407	0.801	−0.358	0.871	−0.341	0.853	−0.696
	*	-	**	-	**	-	**	*
**SCDW**	0.260	−0.193	0.414	−0.005	0.516	0.109	0.880	−0.630
	-	-	-	-	-	-	**	-
**ZDR Z1**	0.433	−0.536	0.331	0.034	0.452	0.068	0.487	−0.526
	-	-	-	-	-	-	-	-
**ZDR Z2**	0.891	−0.311	0.695	−0.328	0.747	−0.099	0.880	−0.662
	***	-	*	-	*	-	**	-
**ZDR Z3**	0.8032	0.147	0.598	−0.143	0.693	0.030	0.795	−0.453
	**	-	-	-	*	-	*	-
**CDS Z1**	0.259	0.010	0.340	0.019	0.604	0.011	0.8182	−0.576
	-	-	-	-	-	-	**	-
**CDS Z2**	0.425	−0.079	0.543	−0.442	0.704	−0.425	0.863	−0.711
	-	-	-	-	*	-	**	*
**CDS Z3**	0.434	−0.255	0.551	−0.344	0.565	−0.476	0.659	−0.491
	-	-	-	-	-	-	-	-
**OS**	-	-	-	-	0.179	−0.446	0.390	−0.606
	-	-	-	-	-	-	-	-
**SMIS**	0.341	0.004	0.611	−0.153	0.582	−0.204	0.860	−0.433
	-	-	*	-	-	-	**	-
**MJCR**	0.621	0.114	0.279	0.0609	−0.190	−0.076	0.450	−0.546
	-	-	-	-	-	-	-	-
**GPT**	0.431	−0.106	0.592	−0.124	−0.007	0.142	0.745	−0.354
	-	-	-	-	-	-	*	-

*P <0.05, **P <0.01, ***P <0.01.

Pearson’s correlation analysis between Knee-Bend score or %TIPPI and knee diameter or the histopathology score parameters at one and six weeks for 250 U and 500 U collagenase-injected animals. CDS, Cartilage degeneration score; CMLW, Cartilage matrix loss width; GPT, Growth plate thickness; MJCR, Medial joint capsule repair; OS, Osteophytes score; SCDW, Significant cartilage degeneration width; SMIS, Synovial membrane inflammation score; TCDW, Total cartilage degeneration width; ZDR, Zonal depth ratio.

### TRPV1 expression

**Figure 8:**
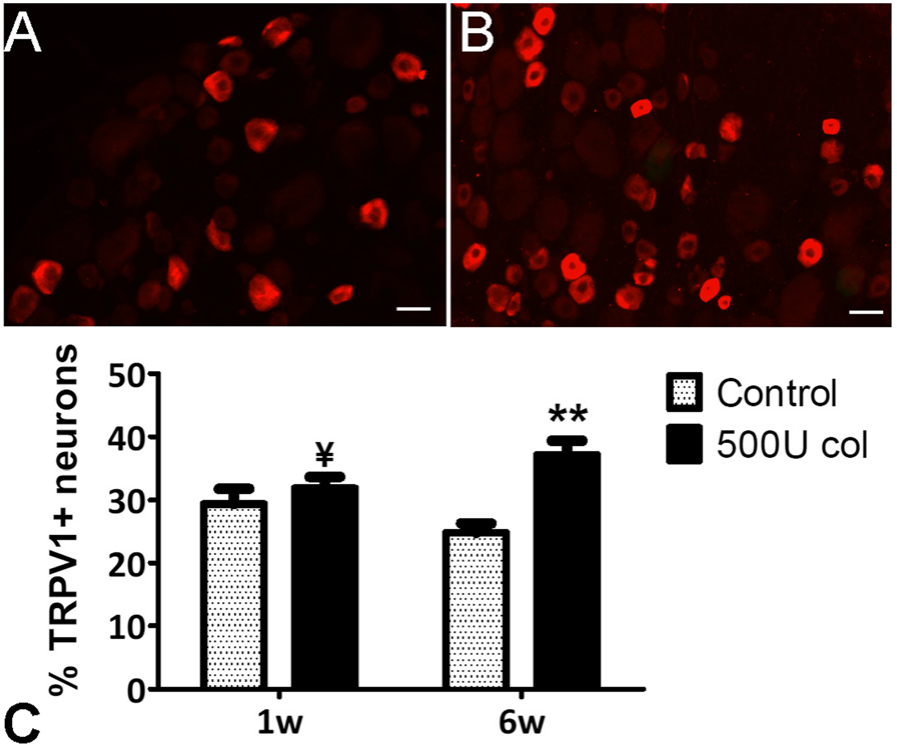
**TRPV1 expression.** Transient receptor potential vanilloid 1 (TRPV1) expression was evaluated in the L3, L4 and L5 ipsilateral dorsal root ganglia (DRG) of control and 500 U collagenase injected rats. **(A, B)** TRPV1 expressed in small-sized L4 DRG neurons of control rats **(A)** and 500 U collagenase-injected rats **(B)**, six weeks after injection. **C:** Six weeks after the injection of 500 U of collagenase there is an increase in the expression of TRPV1. Mean ± SEM. **P <0.01, significantly different from control rats at the same time-point. ^**¥**^*P* <0.05, significantly different from control rats at a different time-point.

Expression of the ion channel TRPV1 was observed in small-sized neuronal cell bodies (Figure 8A, B), as is described for this receptor [39]. In control animals one week after saline injection, 29.4 ± 2.4% of DRG neurons expressed TRPV1 (Figure 8C). At the same time-point, animals injected with 500 U collagenase expressed TRPV1 in 31.9 ± 1.8% of DRG neurons (Figure 8C). Six weeks after saline injection, 25.0 ± 1.4% of DRG neurons were immunoreactive for TRPV1. In contrast, collagenase injected rats showed a significantly increased expression of TRPV1, with a value of 37.1 ± 2.2% positively labelled neurons (*P* <0.01, Figure 8C). Although TRPV1 expression in 500 U collagenase-injected rats at one week was not significantly different from controls at the same time-point, a significant difference was obtained from controls at six weeks (*P* <0.05).

## Discussion

A clinically-relevant model of pain in OA should not only induce relevant nociceptive behaviour but also mimic as closely as possible the structural articular changes observed in the human disease. Here, we show that the injection of type II collagenase into the knee joint of rats leads to the development of histopathological alterations similar to those described for human OA [29], as had been observed in studies in the knee joint of mice [19,40] and rabbits [20], and in the lumbar facet joint of rats [21]. More importantly, we also show for the first time that collagenase-injected rats have nociceptive behaviours associated with movement and loading on the OA joint which represent patients’ major complaints [2].

Focal damage of the articular cartilage, centred on load-bearing areas, is an important pathological trait of human OA [41]. Accordingly, we observed that in 500 U collagenase-injected joints the destruction of the articular cartilage was not homogeneous, mainly affecting the MTP, where extensive areas of the articular cartilage were affected by degenerative changes. The areas of deeper degeneration were always observed in the central part of the MTP as evidenced by the higher ZDR of lesions in zone 2, where, on average, 79% of the cartilage depth was affected. These values are in line with the CDS for each zone, which also showed that the central area of the MTP was the most severely affected. Bone alterations are also an important characteristic of OA. Here, we observed the development of osteophytes, particularly for the 500 U dose after six weeks. Changes in the bone-cartilage interface were also found, with cartilage collapse into the epiphysis being the most obvious.

Another important feature of human OA is its slow development. Although a model that allows a fast development of a disease is favourable for experimental studies, a balance between an adequate experimental time and a rate of disease progression that allows a closer reproduction of the clinical progression of OA is desirable. In the collagenase injection model there is a slow and continuous evolution of the disease. The first relevant signs of cartilage damage were only observable two weeks after injection, and changes consistent with the development of mild OA were first observed at the fourth week. Significant alterations matching the description of human OA [29] were observed after six weeks. In MIA-induced OA, the currently most used model of OA pain, the immediate histopathological effects are more dramatic, leading to total loss of chondrocytes just two weeks after injecting 2 mg of MIA [42]. Neither the initiating events nor many of the pathological changes are typical of OA [14]. Fast chondrocyte depletion is a major disadvantage of the MIA model, since chondrocytes actually play a key role in OA development [43-45]. In fact, it has been shown that little transcriptional similarity exists between rat MIA-induced OA and human OA-derived cartilage [46], probably due to the extensive chondrocyte death associated with the fast progression of OA in the MIA model. So, even though robust nociceptive responses can be quickly obtained with the MIA model, its structural progression does not reproduce OA-like changes as satisfactorily.

One of the key steps in cartilage breakdown during human OA is the loss of type II collagen, characteristic of articular cartilage and one of the main macromolecules of its extracellular matrix [26-28]. Its degradation occurs through the action of matrix metalloproteinases (MMP) with collagenase action, which can be produced by chondrocytes [47]. Accordingly, MMP-13 is overexpressed in OA patients [48] and research has been focusing on the possibility of using MMP inhibitors for OA treatment [49-51]. Additionally, transgenic mice expressing MMP-13 in the articular cartilage develop structural changes very similar to human OA [52], and increased type II collagen degradation was observed in spontaneous OA in Hartley guinea-pigs [53]. In the collagenase model there is a better correlation with what has been described for human OA and spontaneous models of OA, since collagenase can also digest collagen from the extracellular matrix of articular cartilage [20], rendering it susceptible to degradation.

Joint laxity is a well-known risk factor for the development of OA in humans [23-25]. It has been shown that i.a. injection of collagenase leads to increased joint laxity [22] probably as a consequence of the degradation of collagen from menisci and articular ligaments, suggesting that, in this model, OA develops as a consequence of joint instability [18,40], therefore reproducing an important trait of human OA. Surgical models of OA can also induce histological and behavioural alterations as a consequence of joint instability [7,8,54]. However, it is necessary to open the joint capsule and cut the ligaments to induce instability, involving an aggressive destruction of joint structures, reproducing a subset of OA developing as a consequence of traumatic knee injury. In the collagenase model, joint instability is induced by i.a. injection, a simple, non-aggressive method, with little side-effects, which is an advantage of this model.

It seems that the collagenase model of OA can be a relevant alternative model for the study of OA, with a closer reproduction of its cause, its rate of progression and its structural changes. Since our main objective was to evaluate the applicability of a structurally relevant model of OA to study OA-associated pain, we evaluated whether it induces relevant nociceptive changes by assessing nociception associated with movement and loading on the knee joint. We found that both doses of collagenase induced a significant increase in the nociceptive behaviour, with the 500 U dose better correlating with the histopathological changes.

Movement-induced nociception peaked during the disease onset. This was probably due to an inflammatory reaction to collagenase injection [18-20], as is suggested by the increase in the ipsilateral knee diameter one week after collagenase injection, which significantly correlates with the nociceptive changes. Furthermore, both doses of collagenase led to an increase in the synovial membrane inflammation score. This increase was more pronounced with the 500 U dose, with a significant correlation being observed between the degree of synovial membrane inflammation and the nociceptive behaviour. The inflammatory cells infiltrating the synovial membrane will most likely release pro-inflammatory mediators that may act on the primary afferent nerve endings innervating the synovial membrane. This reaction may explain the observed increase in nociception at early time-points. This hypothesis is sustained by the observation that the NSAID diclofenac significantly diminished the nociceptive behaviour at this time point, losing its efficacy at later time-points, when synovial membrane inflammation has receded.

At the sixth week, when the disease is fully established, movement-induced nociception was significantly different from controls in both nociceptive tests. At this time-point, a significant correlation between nociception induced by movement and loading on the joint and the degree of articular changes was also evident with the 500 U dose. The Knee-Bend test highly correlated with both the width and depth of lesions, as well as with the overall degeneration assessed by the CDS. In the Knee-Bend test there is an induced movement of the affected joint and rats’ reactions are scored. In the CatWalk test, on the other hand, changes observed are due not only to nociception arising from loading on the affected knee joint, but also reflect their protective behaviour of placing less weight on the affected joint. This difference could probably account for the less robust correlation seen between this test and the histological scores. Nevertheless, a significant correlation is observed between the %TIPPI for the 500 U dose and the CMLW at 0% depth and the TCDW, as well as with the CDS in zone 2, the most affected area. The magnitude of nociceptive changes was higher for the 500 U dose, even though they were not significantly different from those induced by the 250 U dose. Nevertheless, histopathological changes were much more pronounced for the 500 U dose and had a higher correlation with the behavioural data. Hence, although the 250 U dose effectively induces nociceptive changes, it is not as effective in inducing structural changes. Since the 500 U dose of collagenase induced a set of relevant osteoarthritic-like degenerative changes and correlatable movement-induced nociceptive behaviours six weeks after injection, this was the chosen dose for subsequent studies.

Overall, we hypothesize that during the development of the disease in this model there is a balance between nociception arising from an early inflammatory reaction in the first time-points and nociception resulting from the articular damage at later time points. One could speculate that the steady level of nociception reached after the initial time-points can result from a progressive reduction in inflammation that is counterbalanced by increased joint destruction. The behavioural profile of rats injected with collagenase is very similar to what we had previously observed for the MIA model of OA [13]. The main difference was in the magnitude of changes in the CatWalk test, which were less pronounced in the collagenase model, probably due to the fast depletion of articular cartilage and consequent extensive bone exposure that is seen after injection of 2 mg of MIA [42]. To our knowledge, only one study had addressed nociception in the collagenase model in rats [35]. Using the von Frey and Hargreaves tests, the authors showed that 500 U collagenase injection in the knee of rats leads to the development of mechanical allodynia and thermal hyperalgesia. However, both tests measure referred nociception induced by paw stimulation. Here, we used the Knee-Bend and CatWalk tests to evaluate nociception induced by movement and loading on the affected joint, a more clinically-relevant approach to assess OA-related nociception [13], since it reproduces the major complaint of OA patients.

The efficacy of morphine, in a non-sedative dose [7], in reversing the nociceptive behaviours induced by 500 U of collagenase in both tests, demonstrates that these are, in fact, nociception dependent. This is particularly relevant in the CatWalk test, in which the differences in weight distribution between the hind limbs could otherwise be attributed to the induction of joint instability rather than nociception. The fact that morphine can reverse the changes observed in the %TIPPI at the sixth week shows that there is indeed a decreased loading on the affected joint due to nociception.

The effect of the intra-articular injection of the local anaesthetic lidocaine in diminishing the nociceptive behaviour evaluated by the Knee-Bend and CatWalk tests demonstrates that there is nociceptive input arising from the knee joint. Similar results have also been observed by our group in the MIA model of OA [38]. However, since lidocaine did not fully revert nociceptive changes, it is likely that other mechanisms may be involved, such as, for example, ectopic firing of neurons or central sensitization, that are worth being pursued in future studies.

The expression of TRPV1 was evaluated in L3, L4 and L5 DRG, where the majority of the cell bodies of sensory neurons innervating the joint are located [55]. TRPV1 is selectively expressed in nociceptors [39], being a key molecule in nociceptive pathways that can be activated and/or sensitized by a myriad of molecules [33]. It has been associated with nociception arising from both inflammatory and neuropathic conditions [30-32] and an increased expression of this channel has also been reported for the MIA model of OA [56]. Here, we show that there is an increased expression of TRPV1 after injection of collagenase. Interestingly, a slight increase is also observed in control animals one week after saline injection, when compared with six-week controls, which can be explained by the small inflammatory reaction induced by the injection procedure *per se*. Nevertheless, the increase in collagenase-injected animals is higher. One week after collagenase injection, TRPV1 is already up-regulated. This increase is significantly higher than six-week controls, even though it is not significantly higher than the change observed in control rats at the same time-point. Six weeks after collagenase injection, the increase in TRPV1 expression is further enhanced, being significantly higher than control animals at the same time-point, similarly to what happens in the MIA model [56]. TRPV1 up-regulation in this model shows that changes in primary afferent neurons occur after the development of the disease, providing further evidence of the applicability of this model to the study of nociception associated with OA.

## Conclusion

The different induction methods of animal models of OA seem to lead to distinct outcomes in both joint structural damage and nociception. Since OA itself is a highly heterogeneous condition [57], a better understanding of pain in this disease will certainly be obtained more effectively by using distinct animal models. The collagenase model combines the advantage of inducing OA as a consequence of joint instability and degradation of collagen in the articular cartilage, with the methodologically simple i.a. injection. We show that the injection of a dose of 500 U of collagenase in the rat’s knee leads, after six weeks of disease progression, to a set of both histopathological and nociceptive changes that are consistent with the development of OA-like alterations, along with changes in the sensory innervation of the affected joint. We, therefore, conclude that this model may be a relevant alternative for the study of pain in OA, complementing the currently available animal models.

## Abbreviations

%TIPPI: Percentage of total ipsilateral paw print intensity; CDS: Cartilage degeneration score; CMLW: Cartilage matrix loss width; DRG: Dorsal root ganglia; GPT: Growth plate thickness; i.a.: intra-articular; MIA: monoiodoacetate; MJCR: Medial joint capsule repair; MMP: matrix metalloproteinases; MTP: medial tibial plateau; NSAID: Non-steroidal anti-inflammatory drug; OA: osteoarthritis; OS: Osteophytes score; PBS: phosphate-buffered saline; PBST: PBS + 0.3% triton X; p.o.: *per os*; s.c.: subcutaneously; SCDW: Significant cartilage degeneration width; SMIS: Synovial membrane inflammation score; TCDW: Total cartilage degeneration width; ZDR: Zonal depth ratio.

## Competing interests

The authors declare that they have no competing interests.

## Authors’ contributions

SA participated in the conception and design of the study, performed the induction of the model, tissue processing, histopathology, image and data analysis and statistical analysis, and drafted the manuscript. MM participated in the conception and design of the study, carried out all the behavioural tests and participated in data interpretation. TNS participated in the conception and design of the study, performed the immunofluorescence analysis and participated in data interpretation. JCL participated in the conception, design and coordination of the study and in data interpretation. JFG participated in the conception and design of the study and in data interpretation. FLN participated in the conception and design of the study and in data interpretation, coordinated the study and drafted the manuscript. All authors read and approved the final manuscript.
